# Maize-soybean intercropping improved maize growth traits by increasing soil nutrients and reducing plant pathogen abundance

**DOI:** 10.3389/fmicb.2023.1290825

**Published:** 2023-11-30

**Authors:** Meiyu Liu, Huicheng Zhao

**Affiliations:** ^1^Key Laboratory of Agricultural Water Resources, Center for Agricultural Resources Research, Institute of Genetics and Developmental Biology, Chinese Academy of Sciences, Shijiazhuang, China; ^2^University of Chinese Academy of Sciences, Beijing, China

**Keywords:** maize-soybean intercropping, rhizosphere, microbial community, microbial function, maize growth traits

## Abstract

**Introduction:**

Maize (*Zea mays* L.)–soybean (*Glycine max* L.) intercropping has been widely utilized in agricultural production due to its effectiveness in improving crop yield and nutrient use efficiency. However, the responses of maize rhizosphere microbial communities and the plant pathogen relative abundance to maize growth traits in maize-soybean intercropping systems with different chemical nitrogen fertilizer application rates remain unclear.

**Methods:**

In this study, a field experiment was conducted, and the bacterial and fungal communities of maize rhizosphere soils in maize-soybean intercropping systems treated with different N fertilization rates were investigated using Illumina NovaSeq sequencing. Maize growth traits, soil physicochemical properties and soil enzyme activities were also examined.

**Results and discussion:**

We found that intercropping and N fertilizer treatments strongly influenced soil microbial diversity, structure and function. The PLSPM (partial least squares path modeling) confirmed that soil nutrients directly positively affected maize biomass and that intercropping practices indirectly positively affected maize biomass via soil nutrients, especially NH_4_^+^-N. Intercropping agronomic approaches also improved maize growth traits by reducing the plant pathogen abundance, and the relative abundance of the plant pathogen *Trichothecium roseum* significantly decreased with intercropping treatments compared to monocropping treatments. These results confirmed the benefits of maize-soybean intercropping treatments for agricultural production.

## 1 Introduction

Appropriate cropping systems can improve land use efficiency and increase grain yield per acre. Maize (*Zea mays* L.)–soybean (*Glycine max* L.) intercropping practices can efficiently absorb and utilize nutrient soil resources, regulate the land and resource use efficiency of crop production, and significantly improve the nitrogen use efficiency and photosynthetic efficiency of maize (Martin-Guay et al., 2018; Tilman, 2020; Zhang et al., 2020; Nasar et al., 2021). This system has achieved good economic, social and ecological benefits in many regions (Xia et al., 2013; Sun et al., 2018; Chai et al., 2021). However, more cultivated land, especially mismanaged cropland, still maintains long-term monoculture planting habits and often improves yields with chemically synthesized nitrogen fertilizers, which is also associated with environmental pollution, such as land degradation, water pollution and biodiversity loss (Ju et al., 2009; Guo et al., 2010; Ju and Zhang, 2017). Developing more reasonable crop intercropping methods can not only solve the environmental problems caused by long-term overapplication of N fertilizers but also be beneficial in addressing future challenges (Tilman, 2020; Ryan, 2021).

The maize-soybean intercropping system could increase crop nutrient uptake and yield because soybean roots could secrete more protons and organic acids and activate insoluble nutrient conversion to effective forms for maize absorption (Song et al., 2022). Additionally, maize-soybean intercropping systems utilize soybean biological nitrogen fixation (BNF) to reduce chemical nitrogen fertilizer inputs and alleviate environmental pollution problems (Du et al., 2020). It is inferred that soil nutrient use efficiency is related to maize yield and the maize rhizosphere microecological environment, especially the microbial structure and function in maize rhizosphere soil. Scholars have performed many studies on the effects of monocropping and long-term fertilization on the ecological environment, microbial structure and function (Zhang et al., 2010; Chen et al., 2019), which have greatly contributed to revealing the changes in rhizosphere microecology and nutrients in crop monocropping systems. However, there are few reports on the relationship between maize rhizosphere soil microbial structure and function, soil enzyme activity and maize production in maize-soybean intercropping systems.

Focus on soil microbial structure and function characteristics under different N fertilizer application rates in intercropping systems to understand soil nitrogen cycling and improve nitrogen use efficiency in intercropping systems. The plant rhizosphere contains a rich diversity of microorganisms, and plants benefit from many of them through pathogen invasion suppression and help with acquiring nutrients from the soil in the rhizosphere environment (Nannipieri et al., 2003), which promotes plant growth and maintains soil health (Fierer et al., 2021). In agroecosystems, there is a strong relationship between soil microbial communities and fertilization and a variety of other agricultural management factors (Poulsen et al., 2013; Hartmann et al., 2015; Chen et al., 2019; Wang et al., 2020). Intercropping systems could improve the soil enzyme activities of urease, phosphatase, and invertase, which catalyze the turnover process and increase the contents of soil nutrients for plant growth and utilization (Gong et al., 2019; Sun et al., 2022a). Soil enzyme activities are also closely related to soil physicochemical properties, nutrients, soil microbial diversity, and maize yield (Ablimit et al., 2022). Different cropping patterns strongly influence soil microbes, microbial functions and construction diversity. However, we still lack comprehensive knowledge on how cropping practices, especially maize-soybean intercropping systems, influence crop growth traits and control soil nutrients to shape rhizosphere microbiome structure with multiple metabolic functions.

Long-term chemical N fertilizer application and monoculture of specific crops could increase the abundance of soil fungal plant pathogens, which may cause the occurrence of soil-borne disease in agro-ecosystems (Ratnadass et al., 2012; Zhou et al., 2016). Rhizosphere microbes often inhibit or directly kill pathogens in a variety of ways, thus leading to a reduction in soil-borne disease occurrence. Previous study has demonstrated that maize-soybean intercropping recruits beneficial bacteria in the rhizosphere bacterial community to suppress *Fusarium* (Chang et al., 2022). Phenolic acids were also enriched in root exudates of maize-soybean intercrops to control soil-borne diseases (Gao et al., 2014). Numerous pathogenic species can be harbored in maize (*Zea mays* L.), which could cause severe diseases in maize and negatively affect the yield and quality of the maize crop. Therefore, it is necessary to determine which pathogenic species could affect maize yield and quality in maize-soybean intercropping systems.

To improve the properties and sustainability of agricultural soils, agronomic approaches deserve attention, and many have been implemented. The effects of maize-soybean intercropping systems on soil properties and plant growth have been widely studied (Tilman, 2020; Ryan, 2021). However, little information is available on the effects on soil microbial ecology in the maize rhizosphere. In this study, (i) the influence of intercropping and N fertilization on maize growth traits, soil nutrient levels and soil enzyme activity of the maize rhizosphere under different N application rates in maize-soybean intercropping systems were compared, and (ii) shifts in microbial composition and function were predicted to reveal the regulatory mechanism of rhizosphere microecological effects in maize-soybean intercropping systems.

## 2 Materials and methods

### 2.1 Experimental design

**FIGURE 1:**
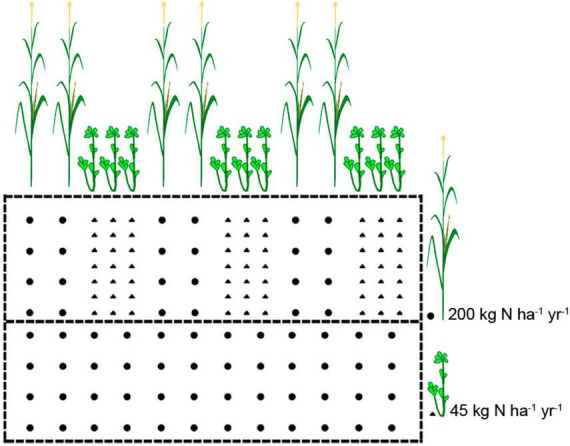
Schematic representation of field experimental setups in this study. The cropping patterns consisted of monocropping maize and its intercropping systems with soybean. Circles represent maize, and triangles represent soybean.

For the experimental setup, maize-soybean intercropping was conducted at the Luancheng Agro-Ecosystem Experimental Station of the Chinese Academy of Sciences, Hebei Province, China (37°53′N, 114°41′E). The soil type in this region is fluvo-aquic soil. The tested maize variety was ZD 958, and the soybean variety was Jidou 37. Four types of treatments were conducted with three replicates in a completely randomized block design, including the following: (1) IN: maize-soybean intercropping system with chemical nitrogen (N) fertilizer application (maize: 200 kg N ha^–1^ year^–1^, soybean: 45 kg N ha^–1^ year^–1^) (2) ICK: maize-soybean intercropping system (unfertilized, 0 kg N ha^–1^ year^–1^), (3) MN: maize monocropping system with chemical N fertilizer application (maize: 200 kg N ha^–1^ year^–1^), and (4) MCK: maize monocropping system (unfertilized, 0 kg N ha^–1^ year^–1^). Each plot was 6 m × 10 m in width and length, and only urea fertilizer was used, without K or P fertilizer. The cropping system was two crops a year, and the experiment was applied after the wheat harvest. Maize and soybean were sown on 25 June 2022 and harvested on 10 October 2022. The urea fertilizer for maize and soybean was applied at the V12 growth stage (large trumpet period) of maize. Monocropping was only planted with a maize row spacing of 60 cm. Maize-soybean intercropping uses a row spacing of 40 cm for soybeans and a row spacing of 60 cm for maize and soybeans. The planting patterns of maize and soybean were shown in Figure 1.

### 2.2 Rhizosphere soil sampling

Maize rhizosphere soil samples from the maize-soybean intercropping experimental setups were collected at the R1 growth stage (silking stage) of maize on 19 September 2022. The maize was carefully uprooted and shaken to remove loosely attached soil around the roots in the field, and the roots were immediately transported to the laboratory. We brushed off the rhizosphere soil adhering to the roots (0–5 mm) and taken as one rhizosphere soil sample (Zhang et al., 2020). Each rhizosphere soil sample was mixed, and impurities such as roots and straw were removed by passing it through a 2 mm sieve. Each soil sample was divided into three parts to store. One part was stored at −20°C for DNA extraction, another part was stored at 4°C to analyze soil ammonium nitrogen (NH_4_^+^-N) and nitrate nitrogen (NO_3_^–^-N) contents within 1 week, and the remaining part was air dried for 10 d to measure other soil physicochemical properties and soil enzyme activities.

### 2.3 Maize growth traits

Maize plants were collected at the silking stage to measure the growth traits of maize, including aboveground biomass, stem and leaf dry mass, and spike weight. The other maize growth traits, including grain weight, axle weight, and ear length, were measured at the maturity stage.

### 2.4 Rhizosphere soil physicochemical properties and enzyme activities

Soil properties were measured as described in previous study (Zhao et al., 2023). Soil NO_3_^–^-N and NH_4_^+^-N were extracted using 2.0 M KCl and determined by spectrophotometry using an ultraviolet photometer (UV-6100S, Shanghai, China). Soil pH was measured with a soil: water ratio of 1: 5 (dry weight/volume) by a pH meter (Mettler-Toledo FE28, Switzerland). Soil total carbon (TC) and dissolved organic carbon (DOC) were determined using a carbon and nitrogen analyzer (Multi N/C 3100, Jena, Germany). Soil total nitrogen (TN) was measured by the Kjeldahl digestion method. Total phosphorus (TP) in the soil was determined by molybdenum blue colorimetry.

Soil enzymes, including urease, sucrase and phosphatase, engage in biochemical reactions and catalyze the turnover process of soil nutrients in ecosystems. Therefore, soil enzyme activities were studied to estimate the contents of soil nutrients (Gong et al., 2019). Soil urease activity was measured using the phenol-sodium hypochlorite colorimetric method at a wavelength of 578 nm (Gu et al., 2009). Sucrase activity was measured by the 3,5-dinitrosalicylic acid colorimetric method using the absorption at 540 nm (Zheng et al., 2022). Soil alkaline phosphatase activity was measured as p-nitrophenol (PNP) released by phosphatase activity after soil incubation with buffered (pH 11.0 for alkaline phosphatase) sodium p-nitrophenyl phosphate solution (115 mM) by spectrophotometry at 405 nm wavelength (Li et al., 2021).

### 2.5 Rhizosphere soil total DNA isolation and molecular analysis

Maize rhizosphere soil total DNA was extracted using the Fast@DNA SPIN Kit (MP Biomedicals, Santa Ana, CA, United States). A portion of 0.5 g of rhizosphere soil was processed according to the manufacturer’s instructions with an additional bead-beating step using a cell homogenizer (BioSpec, Bartlesville, OK, USA 74005) to achieve harsh cell lysis. Extracted DNA was stored at −20°C before further analysis. The primers 515f/806r (5′-GTGYCAGCMGCCGCGGTAA-3′/5′-GGACTACNVGGGTWTCTAAT-3′), targeting the 16S rRNA gene V4 region (Apprill et al., 2015; Parada et al., 2016), and ITS1f/ITS2 (5′-CTTGGTCATTTAGAGGAAGTAA-3′/5′-GCTGCGTTCTTCATCGATGC-3′), targeting the fungal internal transcribed spacer (ITS) region (White et al., 1990; Gardes and Bruns, 1993), were used for amplicon sequencing. PCR amplification was conducted in a 25 μL reaction system using Q5^®^ High-Fidelity DNA Polymerase kits (NEB, #M0491 L) under the following conditions: initial denaturation at 98°C for 2 min, 30 cycles of 98°C for 15 s, 1 min at 55°C for annealing, and 72°C for 30 s, and a final extension at 72°C for 5 min. The amplicons were subjected to Illumina paired-end sequencing on the NovaSeq 6000 platform at Personal Biotechnology Co., Ltd. (Shanghai, China).

### 2.6 Bioinformatic analysis

Bioinformatic analyses were mainly performed with Quantitative Insights Into Microbial Ecology (QIIME 2) (Bolyen et al., 2019). First, the adaptor was cut off with cutadapt (Martin, 2011), and then the dada2 method was used to denoise the paired-end reads (Callahan et al., 2016) and filter out noisy sequences, remove chimeric sequences, join denoised paired-end reads, and then dereplicate those sequences. The resulting amplicon sequence variant (ASV) taxonomy was assigned with Qiime2′s q2-feature-classifier plugin (Bokulich et al., 2018) against the Silva database (version 138) (Quast et al., 2012) for the bacterial 16S rRNA gene and the UNITE database (Nguyen et al., 2016) for the fungal ITS region. The Shannon and Simpson indices were calculated by QIIME 2 (Bolyen et al., 2019). Bacterial community functional profiles were annotated using Functional Annotation of Prokaryotic Taxa (FAPROTAX) (Louca et al., 2016). The ecological guilds of fungal communities were assigned using FUNGuild (Nguyen et al., 2016).

### 2.7 Statistical analyses

Statistical analyses and figure drawing were carried out in R (version 3.4.3). The significance of the difference in variables, such as maize growth traits and soil properties between treatments, was assessed by the Spearman correlation analysis using the “dplyr” package (version 1.1.0). Principal coordinate analysis (PCoA) was performed to visualize the community patterns using the “vegan” package (version 2.6.4) based on Bray-Curtis distance. A Venn diagram displayed the similarity and overlap of species composition among treatments using the “VennDiagram” package (version 1.7.3). The functional features of bacterial and fungal communities were shown using the “pheatmap” package (version 1.0.12). The microbial diversity and functional taxa under different treatments compared to MCK by Duncan’s test. The role of a node of the microbial network was characterized by its within-module connectivity (*Z*i) and among-module connectivity (*P*i) (Olesen et al., 2007). Here, to vividly describe the role of hubs, network hubs (*Z*i > 2.5; *P*i > 0.62), module hubs (*Z*i > 2.5; *P*i ≤ 0.62), connectors (*Z*i ≤ 2.5; *P*i > 0.62) and peripherals (*Z*i ≤ 2.5; *P*i ≤ 0.62) were defined on the basis of the threshold value (Shi et al., 2016). Based on the results of the correlation analysis, the PLSPM (partial least squares path modeling) using the “plspm” package (version 0.5.0) (Sanchez et al., 2023) was established to conceptualize the relationships among the soil properties, microbial characteristics, and maize biomass. Goodness of fit (Gof) was used to evaluate the constructed models, and the final model had a Gof value of 0.48, which was greater than the baseline cutoff value of 0.36 (Wetzels et al., 2009).

## 3 Results

### 3.1 Maize growth traits, rhizosphere soil physicochemical properties, and enzyme activities

The maize growth traits are summarized in Table 1. The intercropping approach (IN, ICK) improved maize growth traits, including maize aboveground biomass, stem and leaf dry mass, grain weight, axle weight and ear length (Table 1). Other growth traits, such as maize grain weight, axle weight and ear length, were influenced by chemical nitrogen fertilizer application (IN) at the maturity stage. Maize rhizosphere soil properties were affected by the intercropping agronomic approach and chemical nitrogen fertilizer. Changes in maize rhizosphere soil physicochemical properties are summarized in Table 2. In the chemical nitrogen fertilizer application treatments, the intercropping treatments (IN) significantly increased the TC, TN, and NO_3_^–^-N contents (*P* < 0.05) compared with the maize monocropping treatments (MN). Maize-soybean intercropping treatments significantly increased the contents of DOC, TP and NH_4_^+^-N (*P* < 0.05) in the unfertilized treatments (ICK), although NO_3_^–^-N was significantly decreased in the ICK (*P* < 0.05). In the maize-soybean intercropping system, the contents of TC and NO_3_^–^-N were significantly increased when chemical nitrogen fertilizer was applied, and the DOC and NH_4_^+^-N content was significantly decreased (*P* < 0.05). There was no significant difference in pH, TN and TP between IN and ICK.

**TABLE 1:** Growth traits of maize in different treatments.

Growth stage	Growth traits	IN	ICK	MN	MCK
Silking stage	Aboveground biomass (g)	302.13 ± 53.23a	299.43 ± 9.56a	223.60 ± 2.45b	237.17 ± 40.04a
	SLDM (g)	126.97 ± 51.17b	131.60 ± 15.67a	87.80 ± 9.93b	98.67 ± 25.57b
	SW (g)	173.97 ± 25.43a	167.77 ± 6.73a	135.87 ± 12.21a	137.07 ± 38.53a
Maturity stage	GW (g)	232.02 ± 18.43a	207.43 ± 21.97b	226.34 ± 39.77b	175.29 ± 12.59b
	AW (g)	34.92 ± 4.29a	24.89 ± 5.21b	33.60 ± 7.17b	19.65 ± 2.00b
	EL (cm)	19.77 ± 2.30a	17.40 ± 1.04b	18.60 ± 2.45b	16.67 ± 0.47b

SLDM, stem and leaf dry mass; SW, spike weight; GW, grain weight; AW, axle weight; EL, ear length. Aboveground biomass, SLDM and SW were measured at the silking stage of maize, GW, AW and EL were measured at the maturity stage of maize. Different small letters indicate significant differences among treatments in the same maize growth traits (*P* < 0.05).

**TABLE 2:** Rhizosphere soil physicochemical properties and enzyme activities in different treatments.

Soil characteristic	IN	ICK	MN	MCK
pH	7.76 ± 0.03a	7.72 ± 0.03a	7.69 ± 0.02a	7.69 ± 0.02a
TC (mg/g)	25.76 ± 2.53a	21.49 ± 0.10b	19.66 ± 0.85b	19.99 ± 0.99b
TN (g/kg)	1.91 ± 0.08a	1.66 ± 0.11ab	1.46 ± 0.02b	1.44 ± 0.02b
DOC (mg/kg)	76.96 ± 3.37b	92.38 ± 3.77a	72.85 ± 5.18b	51.96 ± 3.79c
TP (g/kg)	1.24 ± 0.03a	1.29 ± 0.02a	1.17 ± 0.04ab	1.03 ± 0.01b
NO_3_^–^-N (mg/kg)	26.60 ± 4.44a	4.73 ± 0.21c	12.40 ± 0.85b	14.54 ± 3.75b
NH_4_^+^-N (mg/kg)	0.84 ± 0.27b	3.38 ± 1.45a	0.77 ± 0.07b	0.94 ± 0.26b
Urease (ug/d/g)	3161.25 ± 889.26ab	3999.46 ± 151.29a	1594.3 ± 438.68b	2782.75 ± 75.91b
Phosphatase (nmol/h/g)	1240.91 ± 37.35a	1156.23 ± 52.46a	1123.5 ± 142.53a	1168.82 ± 46.47a
Sucrase (mg/d/g)	71.55 ± 2.22a	82.63 ± 7.39a	74.47 ± 8.02a	77.29 ± 5.81a

TC, total carbon; TN, total nitrogen; DOC, dissolved organic carbon; TP, total phosphorus; NO_3_^–^-N, nitrate nitrogen; NH_4_^+^-N, ammonium nitrogen; IN, maize-soybean intercropping system with urea; ICK, maize-soybean intercropping system (unfertilized); MN, maize monocropping system with urea; MCK, maize monocropping system (unfertilized). Different small letters indicate significant differences among treatments in the same soil characteristic (*P* < 0.05).

**FIGURE 2:**
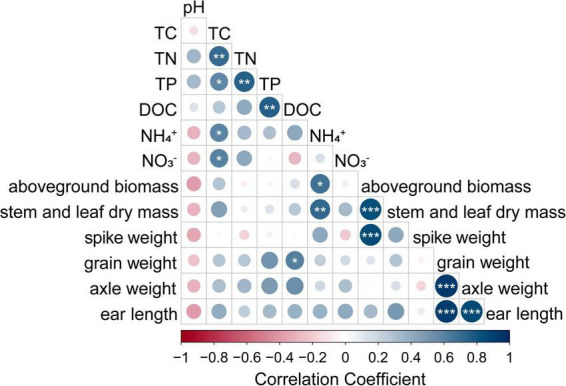
Relationships between maize growth traits and soil physicochemical properties; *indicates a significant correlation determined using Pearson analysis (***P < 0.001; **P < 0.01; *P < 0.05).

According to the results, ICK had the highest NH_4_^+^-N content in all four treatments. Therefore, the maize-soybean intercropping treatments changed the soil nutrient levels; for example, in the maize-soybean intercropping, the soil TN and TC contents increased when N was applied, and the soil TP and DOC contents increased in the unfertilized treatments (Table 2). By using Spearman correlation analysis, the soil NH_4_^+^-N content was positively correlated with maize aboveground biomass and stem and leaf mass (*P* < 0.05) (Figure 2). These results emphasized the close relationship between soybean–maize intercropping and soil nitrogen nutrient levels. The alkaline phosphatase, sucrase and urease activities of rhizosphere soil samples were also presented in Table 2. The maize-soybean intercropping system without fertilization (ICK) significantly increased the activity of urease compared to the other treatments (*P* < 0.05). The highest soil urease activity in ICK may indicate a strong relationship between the intercropping approach and soil nitrogen levels.

### 3.2 Microbial community composition and diversity in maize rhizosphere soil

**FIGURE 3:**
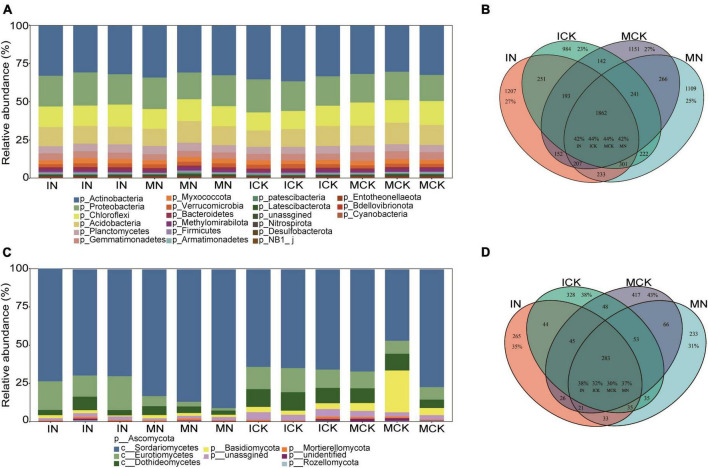
Taxonomic composition of soil bacterial **(A)** and fungal **(C)** communities under the different treatment regimes. Venn diagram showing the distribution of bacterial **(B)** and fungal **(D)** ASVs in different treatments under farmland management regimes. The percentage shows the proportion of the ASV counts in the total number of ASVs. IN, maize-soybean intercropping system with urea; ICK, maize-soybean intercropping system (unfertilized); MN, maize monocropping system with urea; MCK, maize monocropping system (unfertilized).

After sequencing and quality filtering, 1,030,644 clean sequences of the 16S rRNA gene were obtained in total. The most abundant phylum in the bacterial community was Actinobacteria, followed by Proteobacteria, Chloroflexi, Acidobacteria, Planctomycetes, and Gemmatimonadetes. The relative abundance of these 6 phyla accounted for over 87% of the bacterial community (Figure 3A). There were 1,377,489 high-quality ITS sequences used for fungal community analysis, and 64.14% of the total clean reads were assigned to Ascomycota. Within the phylum Ascomycota, 70.40%, 10.80%, and 7.08% of reads were assigned to the classes Sordariomycetes, Eurotiomycetes and Dothideomycetes, respectively (Figure 3C). Basidiomycota was another dominant phylum accounting for 5.09% of the total reads. The bacterial Venn diagram showed that the total number of bacterial ASVs observed in the four treatments was 8521, including 1862 (21.8% proportion in total reads, IN: 42%, ICK: 44%, MCK: 44%, and MN: 42%) common ASVs (Figure 3B). The fungal Venn diagram showed that the fungal ASV total number observed in the four treatments was 1932, including 283 (14.6% proportion of total reads, IN: 38%, ICK: 32%, MCK: 30%, and MN: 37%) common ASVs (Figure 3D).

**FIGURE 4:**
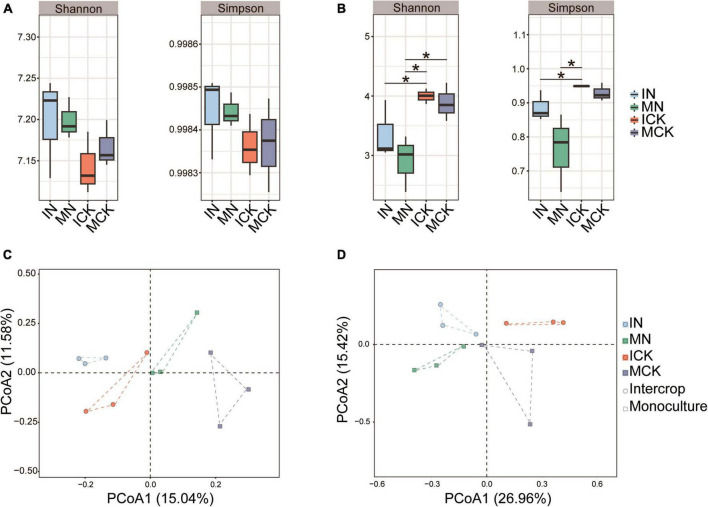
Diversity of soil bacterial **(A)** and fungal **(B)** communities, significant difference in relative abundance compared with control checked by Duncan’s test (*P < 0.05). Principal coordinate analysis (PCoA) ordination of soil bacterial **(C)** and fungal **(D)** communities was performed based on Bray-Curtis distance. IN, maize-soybean intercropping system with urea; ICK, maize-soybean intercropping system (unfertilized); MN, maize monocropping system with urea; MCK, maize monocropping system (unfertilized).

N fertilization had a strong effect on the microbial α-diversity of maize rhizosphere soil, but there were different effects on bacterial and fungal α-diversity (Figures 4A, B). The bacterial Shannon and Simpson indices in both intercropping and monocropping treatments showed an increasing trend with N fertilization, but the fungal α-diversity of maize rhizosphere soil in both intercropping and monocropping treatments was significantly lower than that in the unfertilized treatments. Microbial α-diversity was higher in the maize-soybean intercropping treatments than in the monoculture treatments, although the difference between them was not significant. However, the bacterial community showed lower Shannon and Simpson indices in the rhizosphere soil of the maize-soybean intercropping system without fertilization than in that of the maize monoculture. The PCoA plot showed that both soil bacterial and fungal communities exhibited obvious separation under different treatments (Figures 4C, D). The soil bacterial community changed along the first axis corresponding to different treatments, from intercropping to monocropping. These results indicated that intercropping practices had a strong effect on bacterial communities. In contrast to bacterial communities, soil fungal communities changed along the first axis from N fertilization to no fertilization, indicating the great impact of N fertilization on the soil fungal community. We also found that N fertilization had a negative effect on fungal α-diversity indices.

### 3.3 Prediction of functional profiles for bacterial community and ecological guilds for fungal community composition

**FIGURE 5:**
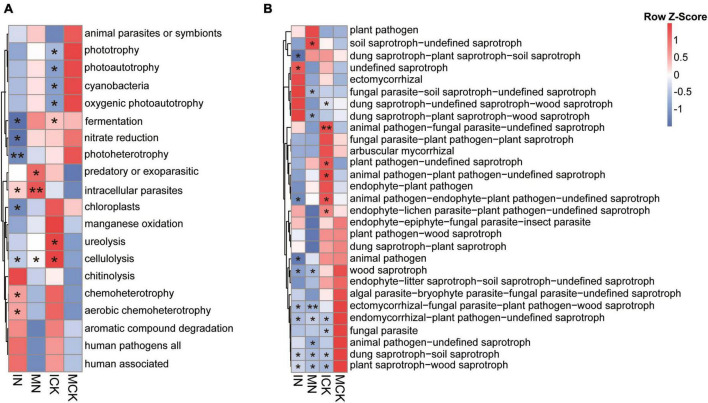
Comparisons of functional community profiles of bacteria **(A)** and fungi **(B)** for different treatments using FAPROTAX (for bacterial community) and FUNGuild (for fungal community). Red squares represent high relative abundance, and blue squares represent low relative abundance. IN, maize-soybean intercropping system with urea; ICK, maize-soybean intercropping system (unfertilized); MN, maize monocropping system with urea; MCK, maize monocropping system (unfertilized). *Indicates a significant difference between treatment and MCK (**P < 0.01; *P < 0.05, determined by Duncan’s test).

Through FAPROTAX (Louca et al., 2016) and FUNGuild (Nguyen et al., 2016), the functions of bacterial taxa and ecological guilds of fungal species were predicted, revealing the soil microbial community functional changes under intercropping and N fertilization regimes (Figures 5A, B). Twenty categories in total were predicted in the bacterial community, the dominant functional groups of the bacterial community were chemoheterotrophy and aerobic chemoheterotrophy, and they were decreased in the MCK treatments. The functions involved in nitrogen cycling, such as ureolysis, were increased by ICK treatments. For example, most of the assigned photosynthetic bacteria were increased in the MCK and MN soils but decreased in the ICK and IN soils. Polysaccharide degradation processes, such as chitinolysis and cellulolysis, were also enhanced in intercropping treatments. In contrast, fungal plant pathogens were the dominant ecological guilds of the fungal community, and the MN soil had the highest relative abundance. This suggested that intercropping decreased the number of plant pathogenic fungi when chemical nitrogen fertilizer was applied, but there was no significant difference between the ICK and MCK treatments. The functional groups of arbuscular mycorrhizal fungi (AMF) were increased and fungal parasites were decreased in the ICK, ectomycorrhizae were increased in the IN practices. Most of the saprotrophic fungal ecological guilds had different trends in the different treatment soils.

### 3.4 Identified keystone species of the rhizosphere microbial communities

**FIGURE 6:**
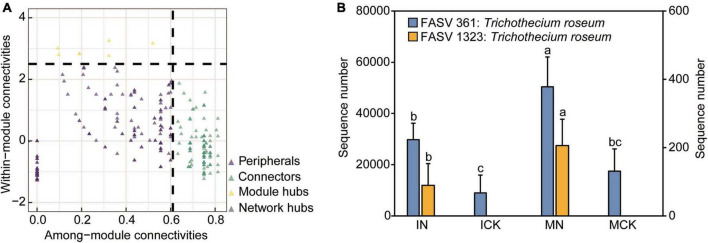
*Z*i-*P*i plot showing the distribution of ASVs based on their network topological roles in the rhizosphere soil **(A)**. The threshold values of *Z*i and *P*i for categorizing ASVs were 2.5 and 0.62, respectively. The relative abundance of keystone species (module hub fungal ASV361, ASV1323) in different treatments **(B)**. IN, maize-soybean intercropping system with urea; ICK, maize-soybean intercropping system (unfertilized); MN, maize monocropping system with urea; MCK, maize monocropping system (unfertilized). Error bars indicate standard error for each treatment in three replicates. Different small letters on each bar indicate significant differences among treatments in the same fungal ASV (*P* < 0.05).

We applied network analysis to identify keystone ASVs using multiple correlations and similarity measures. The *Z*i-*P*i plot showed the distribution of nodes based on their topological roles in aggregate-related networks (Figure 6A). We sorted all species into four subcategories: peripherals, connectors, module hubs, and network hubs. There were 142 nodes assigned to the connectors. These taxa were expected to mediate species interactions between modules and might be very important for function, allowing the exchange of energy and matter between modules. Most connectors were assigned to Actinobacteria (34 nodes), Proteobacteria (21 nodes), Acidobacteria (16 nodes), Chloroflexi (13 nodes), Gemmatimonadetes (7 nodes), Ascomycota (30 nodes), and Basidiomycota (5 nodes). Seven nodes were categorized as module hubs that were particularly strongly interdependent with many nodes in their own modules, and these taxa were expected to mediate species interactions within modules.

In these module hubs (generalists), two fungal keystone nodes (fungal ASV361 and ASV1323) were assigned to *Trichothecium roseum*, which is known to be a mycoparasite and belongs to a common genus of mold that occurs throughout the world. Key fungal ASV361 nodes were significantly depleted in the ICK treatments and significantly enriched in the MN treatments, which indicated that intercropping treatments may significantly decrease soilborne disease occurrence. Nitrogen input treatments increased the opportunity for pathogenic bacteria to encroach on corn plants, and key fungal ASV1323 nodes appeared only in the nitrogen input treatments (Figure 6B). The relative abundance of fungal ASV361 in IN was lower than that in MN, indicating that intercropping may reduce the possibility of pathogen infection caused by chemical nitrogen fertilizer application. The other five module hubs (bacterial ASV2412, ASV7092: *Rokubacteriales*; bacterial ASV3053: *Pseudomonas*; bacterial ASV6616: *Nitrosomonadaceae* MND1; bacterial ASV7890: *Lechevalieria*) belonged to the bacterial community. These keystone node-affiliated taxonomic groups appeared to be important for carbon compound degradation, nitrification, phosphorus utilization and plant immunity (Sun et al., 2022b,2023a). Most nodes assigned to the peripherals mean the node had few or no links with other nodes.

### 3.5 Relationships among rhizosphere soil nutrients, plant pathogens, and maize biomass

**FIGURE 7:**
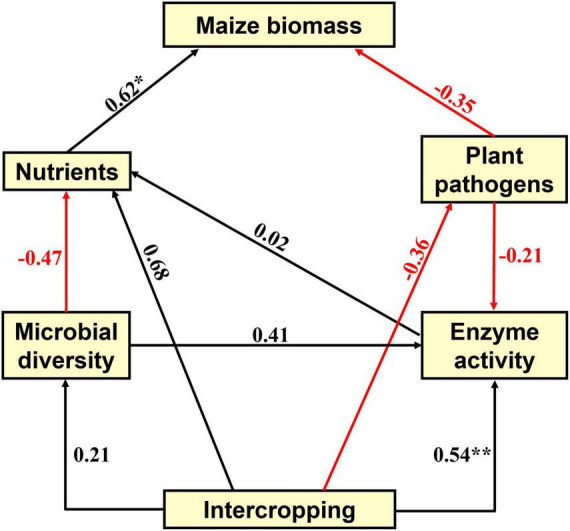
The partial least squares path model (PLSPM) reflects the direct and indirect effects of intercropping treatments on maize growth traits and biomass, soil microbial and physicochemical properties, and soil enzyme activity. Red and black arrows indicate negative or positive effects of variables, respectively, and the numbers associated with the arrows represent the path coefficients. The goodness of fit (Gof) was 0.49. The significance levels are indicated by *P < 0.05 and **P < 0.01.

Soil fertility and microbial community composition have profound effects on plant health (Duchene et al., 2017). To illustrate the effects of intercropping treatments on maize biomass, we performed partial least squares path modeling (PLSPM) analysis to profile the associations among maize biomass, soil nutrients, plant pathogens, microbial diversity and soil enzyme activity (Figure 7). PLSPM analysis results showed that intercropping positively directly affected soil enzyme activity (urease weight 0.66, sucrose weight 0.54, phosphatase weight −0.22) and microbial diversity, but microbial diversity had a negative effect on soil nutrients. Soil nutrients had a strong positive effect (0.62) on maize biomass, suggesting an essential role of soil nutrients in determining maize biomass. Intercropping showed a positive direct effect on soil nutrients (0.68) and a negative direct effect on the relative abundance of potential fungal plant pathogens (−0.36), in turn influencing maize biomass. The modeling analysis results confirmed the importance of soil nitrogen nutrients, particularly NH_4_^+^-N (higher model weight than NO_3_^–^-N), for maize production and suggested that intercropping treatments may improve maize biomass by increasing the nitrogen nutrient level in our investigated soils. The results showed that intercropping increased soil nutrients, reduced plant pathogen damage, and had a potential impact on maintaining high maize yields. As demonstrated above, this model provided a comprehensive understanding of the effects of intercropping systems on maize production and maintained agricultural sustainability for exploring better intercropping agronomic approaches.

## 4 Discussion

Increasing attention has been given to maize-soybean intercropping in agroecology as a means to decrease chemical nitrogen fertilizer inputs. In maize-soybean intercropping systems, many studies have reported nitrogen, phosphorus, and iron nutrition promotion (Xia et al., 2013; Tilman, 2020; Ryan, 2021). In particular, intercropping patterns could improve the nitrogen fixation capacity of legume crops and fix nitrogen for maize uptake (Lai et al., 2022). Rhizosphere microorganisms of maize also play a greater role in the benefits of intercropping systems, and it is important to disentangle the rhizosphere microbiota contributing to the beneficial effects of plants. We compared the maize rhizosphere bacterial communities when cultivated in intercropping and monocropping under different chemical nitrogen fertilizer practices to determine whether the microbial structure differed between the maize plant intercropping and monocropping methods. The distribution of ASVs indicated that both treatments had their own unique species; for the soil microbial community, the presence/absence of species also played a key role in the microbial community composition variation. Therefore, intercropping with soybean could change the microbial community structure in the rhizosphere soil of maize. Our results also highlighted that the rhizosphere bacterial and fungal diversity was improved by maize-soybean intercropping, and the intercropping treatments also played a key role in the composition variation by affecting the presence/absence of species in the rhizosphere soil of maize. This was similar to previous reports that also emphasized the effectiveness of intercropping agronomic practices in improving microbial community diversity (Liu et al., 2021; Xiao et al., 2023).

Microorganisms play an important role in promoting and maintaining agricultural sustainability. The microbial community structure and biodiversity in the rhizosphere soil of intercropped crops are different from those of monocropped crops. Here, we found that some functional groups were identified as keystone nodes, such as ASV6616, which belongs to *Nitrosomonadaceae*, all of whose cultivated representatives are ammonia oxidizers. *Nitrosomonadaceae* generally control nitrification by oxidizing ammonia to nitrite and therefore play major roles in the control of the nitrogen cycle in terrestrial environments. *Nitrosomonadaceae* were key taxa in the maize rhizosphere microbial network associated with intercrop maize cultivars. The abundance of *Nitrosomonadaceae* varies in the maize rhizosphere upon N addition and may promote nitrifiers when maize is intercropped with soybean due to increasing nitrogen. This suggested that intercropping enhanced the activity of nitrogen-cycling microorganisms despite the absence of N fertilization (Meng et al., 2015). Notably, the functional groups of bacteria catalyzing the same processes involved in nitrogen cycling responded differently to intercropping and N fertilization practices. These different environmental adaptations and niche divergence may enhance the persistence of nitrogen cycling in the face of multiple environmental changes, such as long-term fertilization croplands (Sun et al., 2020, 2021, 2023b) and may be an important mechanism for the sustainability of nitrogen cycling.

In the intercropping system, the nutrient N was fixed by soybean and transferred to maize by soil microbes directly or indirectly (Meng et al., 2015). Maize not only obtains nutrients from the soil but also absorbs and uses the fixed nitrogen from bean crops such as soybean. Thus, the nutrients in the soil can be fully utilized by plants, which improves the nutrient utilization rate, and the yield of maize has also been subsequently improved to a certain extent (Ren et al., 2017). Intercropping can also increase dry matter accumulation, possibly by changing the photosynthetic characteristics to be more suitable for maize growth; the larger and more efficient the effective photosynthetic area is, the more adequate the photosynthetic products are to achieve the final high yield of maize (Yang et al., 2017; Nasar et al., 2021). The intercropping system could improve the competitiveness of cereal crops for soil nutrients (He et al., 2023). In this study, most module hub bacterial ASVs belonged to Proteobacteria, Actinobacteria and Methylomirabilota. These species usually showed high heritability in the plant rhizosphere and were considered beneficial for host plant growth and development by producing exopolysaccharide and indole-3-acetic acid and promoting plant iron uptake.

The plant rhizosphere is the key area where soil-borne diseases occur (Gao et al., 2014), and studies have demonstrated that most PGPR can restrict the growth and development of pathogens by producing a variety of antibacterial compounds. In this study, we identified two module hub keystone fungal ASVs as putative keystone species: fungal ASV361 and ASV1323 belonging to *T. roseum*. *T. roseum* is associated with soilborne diseases in microbial community assembly in maize rhizosphere soil, in which germ overwinter in the soil with its mycelium and diseased remains. When the conditions are suitable in the following spring, conidia are produced and spread to the ear, invading through wounds. After the onset of the disease, large numbers of conidia are produced in the affected area, which are spread by wind and rain. Traditional agricultural measures mainly adopt the method of spraying chemical pesticides during the period of pest and disease occurrence, which increases economic costs and poses a certain threat to the environment. Intercropping treatments significantly decreased their relative abundances. Our results indicated that intercropping can significantly reduce the relative abundance of soil disease microorganisms, which to some extent reduces the probability of soil disease occurrence and spread.

Our PLSPM model confirmed that urease had the highest model weight (0.66) on soil enzyme activity compared to sucrose (0.54) and phosphatase (−0.22). In legume and cereal intercropping systems, the activity of urease in soil could be increased (Li et al., 2022). On the one hand, urease can not only catalyze the hydrolysis of urea to ammonia and carbon dioxide but also hydrolyze the fertilizer applied to the soil for plant use. On the other hand, urease can catalyze the hydrolysis of peptide bonds in soil organic compounds. The role of urease in soil is to improve soil nutrients, enhance soil fertility and affect plant biomass or yield. It has been demonstrated that soil enzyme activity is important for improving soil fertility and sustainable agricultural development. Even under lower nitrogen input conditions, the biomass and yield of cereal crops in the intercropping system were still higher than those of monoculture cereal crops. In addition, due to the need for soil nutrients for maize growth, soil nutrients had a strong positive effect on maize growth traits. Although soil microbial diversity had a negative effect on soil nutrients, microbial diversity can positively improve soil enzyme activities, which in turn influence soil nutrients. The benefits of soil enzyme activities in the intercropping system were also reported in the previous study (Gong et al., 2019).

## 5 Conclusion

Through high-throughput sequencing, the impact of intercropping and N fertilizer treatments on microbial communities in maize rhizosphere soil was assessed. We found that maize-soybean intercropping and N fertilization agronomic approaches strongly affected soil bacterial and fungal diversity, soil enzyme activity, and microbial community structure and function. Maize-soybean intercropping treatments significantly decreased the relative abundance of *Trichothecium roseum*, which is associated with soil-borne diseases and may cause plant diseases. The plant pathogenicity properties of the predicted microbial function also diminished in the unfertilized soils, especially in the ICK soil. Nitrogen cycling and polysaccharide degradation functions were also enhanced in the intercropping treatments. The agronomic approach of maize-soybean intercropping improved the aboveground biomass and soil urease activity of maize, which was consistent with the results of the PLSPM model analysis, indicating that maize-soybean intercropping improved soil nitrogen nutrients and reduced plant pathogens to improve maize growth traits.

## Data availability statement

The original contributions presented in the study are publicly available. This data can be found here: NCBI - PRJNA967487.

## Author contributions

ML: Data curation, Investigation, Methodology, Writing – original draft. HZ: Data curation, Funding acquisition, Investigation, Methodology, Resources, Software, Visualization, Writing – original draft, Writing – review and editing, Supervision.
